# HOW DOES SOCIAL DEPRIVATION INFLUENCE END-OF-LIFE FRAILTY TRAJECTORIES? EVIDENCE FROM ELECTRONIC HEALTH RECORDS

**DOI:** 10.1093/geroni/igz038.832

**Published:** 2019-11-08

**Authors:** Daniel Stow, Barbara Hanratty, Fiona E Matthews

**Affiliations:** 1 Institute of Health and Society, Newcastle upon Tyne, United Kingdom; 2 Newcastle University, Newcastle Upon Tyne, England, United Kingdom

## Abstract

Recent research has highlighted inequalities in frailty status, driven by social deprivation over the life course. However, little is known about the interaction between deprivation and frailty over clinically relevant timescales. We used an electronic frailty index, generated automatically in health records at monthly intervals for one year in 13,000 people age >75 who died (cases), matched to 13,000 people with no record of death (controls). We used Index of Multiple Deprivation deciles and latent growth curve models to investigate the impact of deprivation on frailty trajectories. Greater deprivation was associated with higher baseline frailty, but did not influence the rate of change in frailty. We observed greater deprivation driven differences in frailty at baseline in controls than in cases, suggesting that a degree of convergence occurs as people near end-of-life. We discuss these results in light of end-of-life care policy and planning.

